# Treatment of systemic lupus erythematosus patients with the BAFF antagonist “peptibody” blisibimod (AMG 623/A-623): results from randomized, double-blind phase 1a and phase 1b trials

**DOI:** 10.1186/s13075-015-0741-z

**Published:** 2015-08-20

**Authors:** William Stohl, Joan T. Merrill, R. John Looney, Jill Buyon, Daniel J. Wallace, Michael H. Weisman, Ellen M. Ginzler, Blaire Cooke, Donna Holloway, Arunan Kaliyaperumal, Kameswara Rao Kuchimanchi, Tsui Chern Cheah, Erik Rasmussen, John Ferbas, Shelley S. Belouski, Wayne Tsuji, Debra J. Zack

**Affiliations:** Los Angeles County and University of Southern California Medical Center and University of Southern California Keck School of Medicine, 1975 Zonal Ave., Los Angeles, CA 90033 USA; Oklahoma Medical Research Foundation, 825 NE 13th St., Oklahoma City, OK 73104 USA; University of Rochester, 252 Elmwood Ave., Rochester, NY 14627 USA; Hospital for Joint Disease, 301 E 17th St., New York, NY 10003 USA; Cedars-Sinai Medical Center, 8700 Beverly Blvd., Los Angeles, CA 90048 USA; SUNY Downstate Medical Center, 450 Clarkson Ave., Brooklyn, NY 11203 USA; Amgen Inc., 1 Amgen Center Dr., Thousand Oaks, CA 91320 USA; Formerly of Amgen, Inc., 1 Amgen Center Dr., Thousand Oaks, CA 91320 USA; Division of Rheumatology, Los Angeles County + University of Southern California Medical Center and Keck School of Medicine of the University of Southern California, 2011 Zonal Ave., Los Angeles, CA 90033 USA

## Abstract

**Introduction:**

Blisibimod is a potent B cell-activating factor (BAFF) antagonist that binds to both cell membrane-expressed and soluble BAFF. The goal of these first-in-human studies was to characterize the safety, tolerability, and pharmacokinetic and pharmacodynamic profiles of blisibimod in subjects with systemic lupus erythematosus (SLE).

**Methods:**

SLE subjects with mild disease that was stable/inactive at baseline received either a single dose of blisibimod (0.1, 0.3, 1, or 3 mg/kg subcutaneous [SC] or 1, 3, or 6 mg/kg intravenous [IV]) or placebo (phase 1a; N = 54), or four weekly doses of blisibimod (0.3, 1, or 3 mg/kg SC or 6 mg/kg IV) or placebo (phase 1b; N = 63). Safety and tolerability measures were collected, and B cell subset measurements and pharmacokinetic analyses were performed.

**Results:**

All subjects (93 % female; mean age 43.7 years) carried the diagnosis of SLE for ≥ 1 year. Single- and multiple-dose treatment with blisibimod produced a decrease in the number of naïve B cells (24–76 %) and a transient relative increase in the memory B cell compartment, with the greatest effect on IgD^-^CD27+; there were no notable changes in T cells or natural killer cells. With time, memory B cells reverted to baseline, leading to a calculated 30 % reduction in total B cells by approximately 160 days after the first dose. In both the single- and multiple-dosing SC cohorts, the pharmacokinetic profile indicated slow absorption, dose-proportional exposure from 0.3 through 3.0 mg/kg SC and 1 through 6 mg/kg IV, linear pharmacokinetics across the dose range of 1.0–6.0 mg/kg, and accumulation ratios ranging from 2.21 to 2.76. The relative increase in memory B cells was not associated with safety signals, and the incidence of adverse events, anti-blisibimod antibodies, and clinical laboratory abnormalities were comparable between blisibimod- and placebo-treated subjects.

**Conclusions:**

Blisibimod changed the constituency of the B cell pool and single and multiple doses of blisibimod exhibited approximate dose-proportional pharmacokinetics across the dose range 1.0–6.0 mg/kg. The safety and tolerability profile of blisibimod in SLE was comparable with that of placebo. These findings support further studies of blisibimod in SLE and other B cell-mediated diseases.

**Trial registration:**

Clinicaltrials.gov NCT02443506. Registered 11 May 2015. NCT02411136 Registered 7 April 2015.

## Introduction

Systemic lupus erythematosus (SLE) has a heterogeneous clinical and immunologic presentation, with the commonality of B cell hyperactivity across the patient population. B cell-activating factor (BAFF, also known as B lymphocyte stimulator [BLyS]) is crucial to the survival and differentiation of B cells [1–5]. In animal models of SLE, BAFF overexpression promotes features of SLE [6–10] and BAFF antagonists attenuate disease progression [8, 11–13]. In humans with SLE, circulating BAFF levels are increased in as many as 50 % of patients [14–16] and numerous studies have found a positive correlation between BAFF expression and disease activity [17–21].

Given its likely contributory role to the maintenance of SLE disease activity, BAFF is an appealing target in the treatment of SLE. Two phase 3 clinical trials of the antisoluble BAFF monoclonal antibody belimumab added to standard-of-care treatment demonstrated significant effects on disease activity [22, 23]. The “peptibody” blisibimod (A-623, AMG 623) is also a BAFF antagonist, but unlike belimumab, it binds to both membrane-bound and soluble BAFF. Blisibimod inhibits the interaction of BAFF with any of its three receptors (half-maximal inhibitory concentration [IC_50_] = 0.2 nM at 1 nM BAFF), inhibits the in vitro BAFF-driven increases in B cell proliferation (IC_50_ = 0.1 nM at 0.2 nM BAFF), reduces in vivo peripheral B cell numbers in non-autoimmune-prone mice, and delays the onset of proteinuria in SLE-prone (NZBxNZW)F1 mice [24]. Neutralization of both soluble and membrane-expressed BAFF by blisibimod may produce an immunologic change different from that upon neutralization of soluble BAFF alone.

We present the safety, tolerability, and pharmacokinetic and pharmacodynamic profiles of blisibimod in subjects with SLE in two multicenter, double-blind, placebo-controlled trials: a single-dose phase 1a first-in-human study and a multiple-dose phase 1b study.

## Methods

### General

The studies were conducted at 12 sites in the United States. The protocols were approved by each center’s institutional review board (IRB) (please see the Acknowledgements), and all subjects provided written informed consent. The studies were developed and sponsored by Amgen, Inc., and blisibimod is now licensed to Anthera Pharmaceuticals, Inc. (Hayward, CA, USA).

### Subjects

Men and women 18–65 years of age with mild disease that was either stable or inactive (as assessed by the investigator) were eligible for enrollment if they had a diagnosis of SLE for at least 1 year, and met the updated American College of Rheumatology classification criteria [25, 26]. Subjects were excluded if they did not have a current or previous positive antinuclear antibody test; had active vasculitis, central nervous system lupus, acute renal disease, a glomerular filtration rate < 60 mL/min, uncontrolled hypertension or diabetes, or active infection within the previous 30 days; or had antibodies to human immunodeficiency virus, hepatitis B surface antigen, or hepatitis C. Those who were pregnant or nursing were also excluded, as were those who had either received monoclonal antibody therapy as an investigational drug within the previous year or participated in other investigational drug or device trials within 30 days. Subjects who had received cyclosporine, tacrolimus, sirolimus, cyclophosphamide, other alkylating agents, or the equivalent of > 100 mg/day of prednisone (pulse therapy) within 60 days or rituximab within 12 months were also not eligible. However, subjects were allowed to be treated with other SLE medications, including ≤ 10 mg/day of prednisone or its equivalent. Other than the proscribed medications above, investigators were permitted to prescribe concomitant medications or treatments as clinically warranted. Subjects had to be willing to practice effective methods of contraception.

### Study design

In the first-in-human phase 1a ascending single-dose study, subjects were sequentially enrolled into one of seven dose cohorts: 0.1, 0.3, 1.0, or 3.0 mg/kg subcutaneous (SC) or 1.0, 3.0, or 6.0 mg/kg intravenous (IV) blisibimod. The 0.1 mg/kg dose was chosen as the starting dose on the basis of multiple-dosing preclinical toxicology findings in cynomolgus monkeys; the remaining doses were based on preclinical pharmacokinetic modeling and toxicology studies that showed no adverse effects (AEs) at weekly doses up to 150 mg/kg IV or SC [data on file]. Within each dose cohort, subjects were randomized (3:1 ratio) to receive blisibimod or placebo, and were followed for 28 (0.1 and 0.3 mg/kg) or 42 days (1.0, 3.0, and 6.0 mg/kg) post-dose, and those in the highest dose cohort (6.0 mg/kg) completed a 4-week extension follow-up visit after their end-of-study visit. In the phase 1b ascending multiple-dose study, subjects were sequentially enrolled into one of four dose cohorts: 0.3, 1.0, or 3.0 mg/kg SC or 6.0 mg/kg IV. Within each dose cohort, subjects were randomized (4:1 ratio) to receive four weekly doses of blisibimod or placebo, and the pharmacokinetics of blisibimod were followed to day 63 (0.3 and 1.0 mg/kg) or day 112 (3.0 and 6.0 mg/kg). For both studies, blisibimod was given as a 30 mg/mL formulation in vehicle. Those assigned to the placebo group received an equivalent volume of the corresponding vehicle.

### End points

The primary end point of both studies was the characterization of the safety and tolerability of blisibimod. Safety and tolerability assessments included the incidence of treatment-emergent AEs, and clinically significant changes in vital signs, electrocardiograms (ECGs), and clinical laboratory safety tests. AEs were classified using Medical Dictionary for Regulatory Activities (MedDRA) terminology, and clinical laboratory results were assessed using the grading criteria of the National Institute of Health Common Toxicity Criteria for Adverse Events (NIH CTCAE). Secondary end points were the pharmacokinetic and pharmacodynamic profiles of blisibimod following single or multiple SC or IV doses. Exploratory end points included changes in peripheral blood B cell counts and expression of B cell surface markers.

### Pharmacokinetic analysis

Serum concentrations of blisibimod were assessed at each visit beginning on day 1 (the day before dosing) and continuing through the end of the studies, with extensive serum collections after the first and fourth doses (phase 1b study only). Pharmacokinetic parameters were derived for each subject from the blisibimod serum concentrations: these parameters included area under the plasma-concentration curve from time of treatment to the last measurable concentration (AUC_0-t_)_,_ maximum observed serum concentration (C_max_), estimated initial concentration after IV bolus dosing (C_0_), time to C_max_ (t_max_), half-life (t_1/2,z_), clearance (CL), apparent clearance after SC dosing (CL/F), and accumulation ratio (AR). Blisibimod concentrations over time, as well as pharmacokinetic parameter values were summarized by route and dose.

### Immunogenicity analysis

The presence of neutralizing serologic reactivity to blisibimod was analyzed using two validated assays at screening in both studies, at the end of the phase 1a study, and on day 29 in the phase 1b study. The first was a Biacore immunoassay to establish the presence of binding antibodies, and the second was an in vitro cell-based bioassay to detect neutralizing or inhibitory effects toward blisibimod in pre- and post-dose samples. All samples that tested positive in the immunoassay were tested in the bioassay along with their corresponding pre-dose samples, even if the pre-dose samples did not test positive in the immunoassay. If a sample was positive in both the Biacore assay and the cell-based assay, the subject was defined as positive for neutralizing antibodies.

### Exploratory B cell analysis

In the phase 1a study, whole blood specimens were collected at screening visit day 1, pre-dose on the day of dosing, post-dose on days 14 and 28 for all dose groups, day 42 for the 3.0 and 6.0 mg/kg dose groups, and at the extension/follow-up for all dose groups. In the phase 1b study, whole blood specimens were collected twice before study inclusion (at screening day 28, re-screen); pre-dose on day 1; post-dose on days 15, 29, 50, 64, 78, 110, 134, 164, 190, 218; and early termination (when applicable). Whole blood specimens were shipped at ambient temperature to ICON Central Laboratories within the previously established 48-hour stability time frame for standard hematology assessment and flow cytometry determinations [data on file]. A standard clinical hematology analyzer was used to enumerate total lymphocyte counts. Detailed methods are provided elsewhere [27].

Antibody combinations were chosen to permit enumeration of total B cells (CD19^+^CD20^+^), naïve B cells (CD19^+^sIgD^+^CD27^-^), IgD^+^ memory B cells (CD19^+^sIgD^+^CD27^+^), IgD^-^ memory B cells (CD19^+^sIgD^-^CD27^+^), total T cells (CD3^+^CD16/CD56^-^), and natural killer cells (CD3^-^CD16/CD56^+^). Plasmablasts were previously assessed and determined not to be sufficiently stable for assessment in the phase 1 studies [27]; although they were analyzed in the phase 1a study, the data were not used because of cell instability. The antibody configurations and clone numbers were as follows: Tube 1: CD20 FITC (L27), CD45 APC (2D1), CD19 PerCP (SJ25C1); Tube 2: CD3 FITC (SK7), CD16 PE (B73.1), CD56 PE (MY31), CD45 APC (2D1); Tube 3: CD27 FITC (L128), CD38 PE (HB7), CD19 PerCP (SJ25C1), CD45 APC (2D1); Tube 4: CD27 FITC (L128), IgD PE (IA6-2), CD19 PerCP (SJ25C1), CD45 APC (2D1), Tube 5: CD138 FITC (MI15), CD38 PE (HB7), CD19 PerCP (SJ25C1), CD45 APC (2D1). The entire panel was performed in the phase 1a study; in the phase 1b study, the routine panel (tubes 1–3) was performed at screening visit day 28, re-screen, and days 64, 110, 134, 164, and 190. The extended panel (all tubes) was performed on phase 1b specimens drawn on days 1, 15, 29, 50, 78, 218, and early termination. Monitoring was not required if the change in B cell level was ≤ 20 %, or levels had returned to either within 20 % of baseline or a level from a reference range of healthy volunteers, whichever was lower; subjects could be released from monitoring as early as day 190. Those that did not meet these criteria were asked to return monthly until the criteria were met or they were released by medical board review.

### Statistical analyses

Demographic, safety, pharmacokinetic, and B cell data for each route and dose were analyzed with descriptive statistics. After review, data were combined for the placebo groups by any route. The primary safety analysis was performed for all subjects who received an investigational product (blisibimod or placebo).

The relationship between blisibimod dose and changes in lymphocyte phenotypes was explored graphically using a cubic spline [28] to visualize potential trends over time by dose and route. After reviewing these graphs, exploratory modeling of the relationship between dose/route and select lymphocyte phenotypes was analyzed using a mixed-effects model, with baseline value and actual treatment as fixed effects, and subject as a random effect. Based on graphical review, post-dose times were grouped as dosing (days 15–42), post-dosing (days 43–100), and extended follow-up (> day 100) phases, with exploratory modeling performed separately for these three post-dose time ranges. Because they were similar, blisibimod 1.0 mg/kg and 3.0 mg/kg dose groups were pooled and compared with the placebo group for each post-dose phase using an F test (*P* values reported for these comparisons should be considered descriptive). Reference ranges of the mean, standard error, and coefficient of reliability of healthy volunteer and SLE subsets were established in a previously published study [27].

## Results

### Subject disposition and baseline characteristics

In the phase 1a study, 56 subjects were enrolled and randomized; 54 were dosed. Two subjects (blisibimod, n = 1; placebo, n = 1) did not receive the study drug due to study ineligibility: the subject in the blisibimod group developed an infection before dosing, and the subject in the placebo group had a glomerular filtration rate < 60 mL/min. Only one subject (placebo) was withdrawn from the phase 1a study due to a serious adverse event (SAE; see Safety and tolerability). In the phase 1b study, 64 subjects were enrolled and randomized; 63 were dosed. One blisibimod-assigned subject did not receive the study drug due to study ineligibility (papillary lesion requiring an excision), and one placebo-assigned subject who received blisibimod instead of placebo at the fourth dose was excluded from the analyses. Four subjects (blisibimod, n = 2; placebo, n = 2) withdrew from the study due to an AE: in the blisibimod cohort, AEs were migraine and hypersensitivity, and in the placebo cohort, AEs were cellulitis and abscessed limb. One additional subject from the placebo group withdrew from the study at the subject’s request.

Demographics and baseline disease characteristics were similar between the treatment groups, with the exception of CD19 antigen median fluorescence intensity (MFI), which was statistically significantly higher in the blisibimod versus placebo group (Tables 1, 2). Concomitant medication use was high, with a large portion of subjects taking prednisone (39–45 %) and hydroxychloroquine (38–63 %).

**Table 1:** Demographics and disease characteristics of patients with systemic lupus erythematosus in the phase 1a study

		Blisibimod							
	Placebo	mg/kg SC				mg/kg IV			All
Baseline characteristics	(n = 14)	0.1 (n = 6)	0.3 (n = 6)	1.0 (n = 9)	3.0 (n = 6)	1.0 (n = 6)	3.0 (n = 1)	6.0 (n = 6)	(N = 40)
Sex, n (%)									
Female	13 (93)	5 (83)	6 (100)	8 (89)	6 (100)	6 (100)	1 (100)	6 (100)	38 (95)
Male	1 (7)	1 (17)	0 (0.0)	1 (11)	0 (0.0)	0 (0.0)	0 (0.0)	0 (0.0)	2 (5)
Race, n (%)									
White	2 (14)	2 (33)	1 (17)	1 (11)	0 (0)	2 (33)	1 (100)	3 (50)	10 (25)
Black	5 (36)	5 (50)	3 (50)	1 (11)	2 (33)	1 (17)	0 (0)	2 (33)	12 (30)
Hispanic	7 (50)	0 (0)	2 (33)	7 (78)	4 (67)	3 (50)	0 (0)	0 (0)	16 (40)
Asian	0 (0)	0 (0)	0 (0)	0 (0)	0 (0)	0 (0)	0 (0)	1 (17)	1 (3)
Pacific Islander	0 (0)	1 (17)	0 (0)	0 (0)	0 (0)	0 (0)	0 (0)	0 (0)	1 (3)
Age (years)									
Median	39.5	48.5	47.5	46.0	45.0	38.0	52.0	53.0	46.0
Range	21–55	32–53	30–52	31–55	36–53	28–55	--	32–55	28–55
Medications, n (%)									
Prednisone (+ MePred)	7 (50)	3 (50)	1 (17)	7 (78)	4 (67)	3 (50)	1 (17)	0 (0)	18 (45)
Hydroxychloroquine	6 (43)	2 (33)	2 (33)	6 (67)	4 (67)	1 (17)	0 (0)	0 (0)	15 (38)
Azathioprine	4 (29)	3 (50)	1 (17)	3 (33)	1 (17)	1 (17)	0 (0)	0 (0)	9 (23)
Mycophenolate	4 (29)	0 (0)	2 (33)	3 (33)	1 (17)	0 (0)	1 (17)	0 (0)	7 (18)
Methotrexate	1 (7)	0 (0)	0 (0)	1 (11)	0 (0)	2 (33)	0 (0)	0 (0)	3 (8)
Anti-dsDNA, n	12	6	6	9	6	4	5	0	36
Median (range), IU/mL^a^	30 (30–300)	179 (30–300)	30 (30–206)	30 (30–300)	30 (30–187)	30 (30–294)	30 (30–40)	--	30 (30–300)
C3 (IU,mL), median (range)	114 (58–168)	131 (76–189)	132 (104–188)	126 (95–187)	123 (63–154)	148 (95–173)	113 (111–120)	--	125 (63–189)
C4 (IU,mL), median (range)	13 (5–38)	17 (13–40)	34 (12–38)	22 (13–44)	14 (9–30)	33 (23–40)	23 (18–31)	--	23 (9–44)

*Abbreviations*: *C3* complement 3, *C4*, complement 4, *dsDNA* double-stranded DNA, *IV* intravenous, *MePred* methylprednisolone, *SC* subcutaneous

^a^Reference ranges are < 30 IU/mL for negative, 30–75 IU/mL for borderline, and > 75 UI/mL for positive for all age ranges

**Table 2:** Demographics and disease characteristics of patients with systemic lupus erythematosus in the phase 1b study

		Blisibimod				
	Placebo	mg/kg SC			6.0 mg/kg IV	All
Baseline characteristics	(n = 13)	0.3 (n = 12)	1.0 (n = 13)	3.0 (n = 12)	(n = 12)	(N = 49)
Sex, n (%)						
Female	12 (92)	12 (100)	12 (92)	12 (100)	9 (75)	45 (92)
Male	1 (8)	0 (0)	1 (8)	0 (0)	3 (25)	4 (8)
Race, n (%)						
White	6 (46)	7 (58)	9 (69)	4 (33)	5 (42)	25 (51)
Black	2 (15)	3 (25)	3 (23)	1 (8)	2 (17)	9 (18)
Hispanic	5 (38)	1 (8)	1 (8)	6 (50)	5 (42)	13 (27)
Asian	0 (0)	1 (8)	0 (0)	1 (8)	0 (0)	2 (4)
Pacific Islander						
Age (years), median (range)	45.0 (22–64)	41.5 (21–62)	47.0 (34–63)	44.0 (24–66)	44.5 (26–62)	43.0 (21–66)
SLEDAI, median (range)	3 (0–11)	4 (0–6)	2 (0–14)	4 (0–12)	2 (0–10)	2 (0–14)
Medications, n (%)						
Prednisone (+ MePred)	7 (54)	4 (33)	5 (38)	6 (50)	4 (33)	19 (39)
Hydroxychloroquine	13 (100)	4 (33)	9 (69)	9 (75)	9 (75)	31 (63)
Azathioprine	4 (31)	1 (8)	4 (31)	1 (8)	1 (8)	7 (14)
Mycophenolate	2 (15)	2 (17)	1 (8)	1 (8)	0 (0)	4 (8)
Methotrexate	0 (0)	1 (8)	0 (0)	1 (8)	1 (8)	3 (6)
Anti-dsDNA, n	13	12	13	11	12	48
Median (range), IU/mL^a^	36 (30–300)	30 (30–300)	36 (11–300)	30 (30–300)	30 (30–300)	30 (11–300)
C3 (IU/mL), median (range)	96 (47–167)	131 (78–179)	100 (65–266)	107 (74–160)	119 (82–168)	119 (65–266)
C4 (UI/mL), median (range)	20 (9–43)	19 (5–58)	23 (7–55)	21 (8–46)	23 (5–50)	21 (5–58)

*Abbreviations*: *C3* complement 3, *C4* compliment 4, *dsDNA* double-stranded DNA, *IV* intravenous, *MePred* methylprednisolone, *SC* subcutaneous, *SLEDAI* Systemic Lupus Erythematosus Disease Activity Index

^a^Reference ranges are < 30 IU/mL for negative, 30–75 IU/mL for borderline, and > 75 UI/mL for positive for all age ranges

### Blisibimod pharmacokinetics

**Fig. 1:**
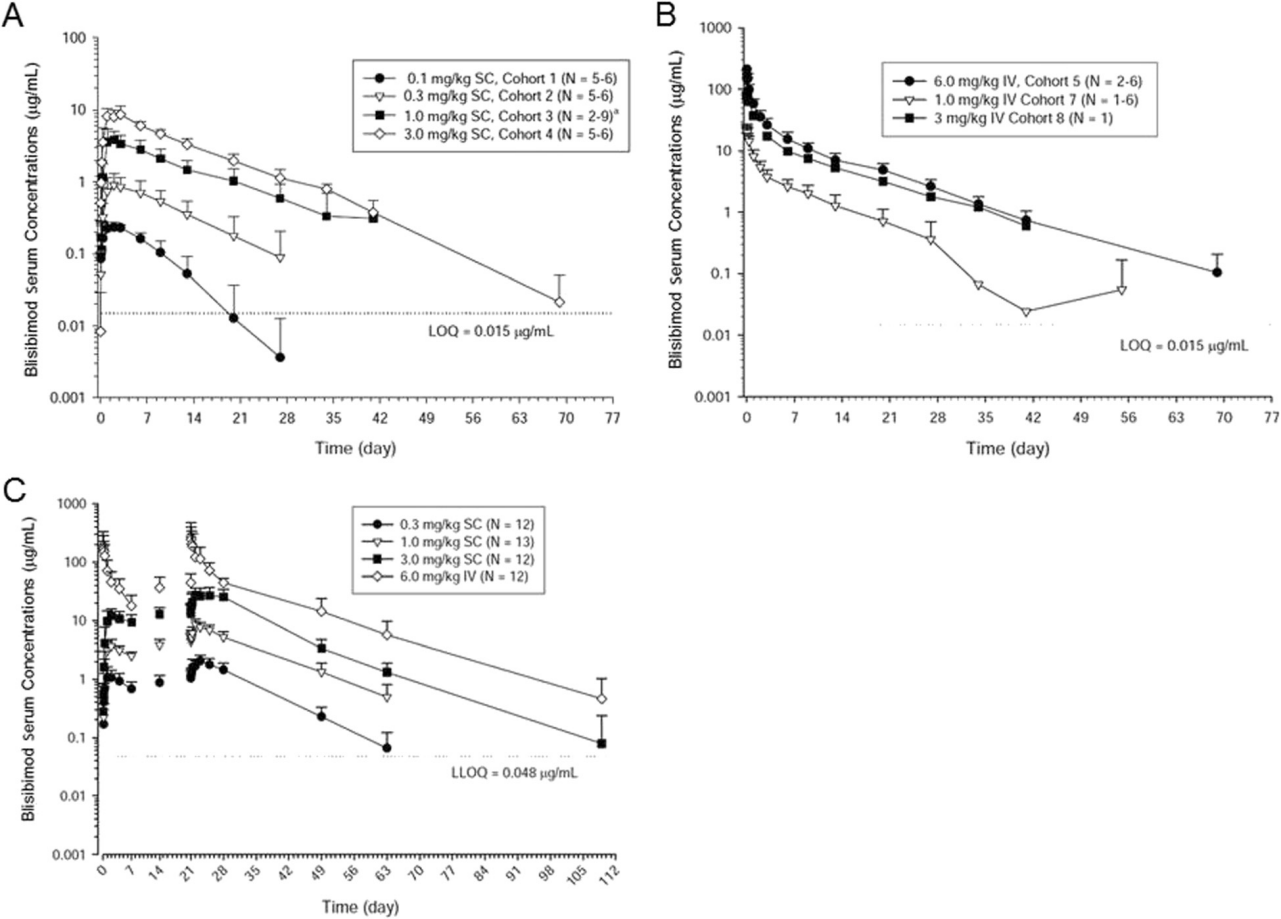
Mean (SD) concentration-time profiles following administration of blisibimod in subjects with systemic lupus erythematosus. Subjects were treated with the indicated single doses of blisibimod IV (**a**), SC (**b**), or the indicated weekly doses (**c**) for 4 weeks. *Abbreviations*: *IV* intravenous, *LOQ* lower limit of quantification, *SC* subcutaneous, *SD* standard deviation

After single and four weekly IV injections, blisibimod serum concentration-time profiles were biphasic: there was an initial distribution phase followed by a slower elimination phase (Fig. 1a, c). Based on C_0_ and AUC, exposure increased in an approximate dose-proportional manner from 1.0 to 6.0 mg/kg IV (Tables 3, 4). In the single-dose cohorts, the mean t_½,z_ for the 6.0 mg/kg IV dose group was 8.7 days (Table 3); in the multiple-dose cohort for the 6.0 mg/kg IV dose group, 12 days (Table 4). The mean CL ranged from 47.5 to 51.9 mL/hr after a single IV dose (Table 3) and 34 mL/hour after multiple IV doses (Table 4). These similar CL values indicate linear pharmacokinetics across a sixfold range of 1.0 to 6.0 mg/kg. Blisibimod exhibited a 2.3-fold AR after the fourth dose compared with the first dose (Table 4).

**Table 3:** Pharmacokinetic parameters and incidence of anti-blisibimod antibodies in patients with systemic lupus erythematosus in the phase 1a study

	mg/kg SC				mg/kg IV		
	0.1 (n = 6)	0.3 (n = 6)	1.0 (n = 9)	3.0 (n = 6)	1.0 (n = 6)	3.0 (n = 1)	6.0 (n = 6)
Parameter, mean (SD)							
t_max_ (hr)	48 (2.2–72)	48 (47–70)	48 (24–72)	47 (23–73)	0.61 ( 0.083–1.1)	0.083	0.42 (0.17–0.67)
C_max_ or C_0_ (μg/hr)	0.273 (0.0817)	0.916 (0.397)	4.14 (1.54)	9.32 (2.57)	21.2 (4.11)	80.9	190
AUC_0-t_ (hr*μg/mL)	50.0 (15.9)	252 (123)	1140 (384)	2770 (459)	1460 (543)	6610	9440 (1980)
t_1/2,z_ (day)	4.0 (1.1)	6.5 (2.9)	9.8 (2.5)	8.4 (2.1)	7.9 (2.8)	8.4	8.7 (2.0)
CL/F or CL (mL/hr)	179 (62.2)	112 (49.4)	72.0 (26.9)	77.8 (24.7)	51.9 (6.96)	48.6	47.5 (7.54)
Antibodies, n (%)							
Pre-dose only	0 (0)	3 (50)	0 (0)	0 (0)	1 (17)	0 (0)	0 (0)
Post-dose only	4 (67)	1 (17)	3 (33)	1 (17)	0 (0)	1 (17)	0 (0)
Pre- and post-dose	0 (0)	0 (0)	0 (0)	2 (33)	0 (0)	2 (33)	1 (100)

*Abbreviations*: *AUC*_*0-t*_ plasma-concentration curve from time of treatment to the last measurable concentration, *C*_*max*_ maximum observed serum concentration, *C*_*0*_ estimated initial concentration after IV bolus dosing, *CL* clearance (CL), *CL/F* apparent clearance, *IV* intravenous, *SC* subcutaneous, *SD* standard deviation, *t*_*1/2,z*_ half-life, *t*_*ma*x_ time to C_max_

**Table 4:** Pharmacokinetic parameters and incidence of anti-blisibimod antibodies in patients with systemic lupus erythematosus in the phase 1b study

	0.3 mg/kg SC (n = 12)		1.0 mg/kg SC (n = 13)		3.0 mg/kg SC (n = 12)		6.0 mg/kg IV (n = 12)	
Parameter, mean (SD)	Day 1	Day 22	Day 1	Day 22	Day 1	Day 22	Day 1	Day 22
t_max_ (hr)	47 (24–97)	48 (24–96)	49 (25–170)	26 (8–73)	47 (20–170)	49 (0.58–170)	N/A	N/A
C_max_ or C_0_ (μg/hr)	1.18 (0.498)	2.13 (0.466)	3.84 (1.02)	8.21 (1.88)	13.2 (4.49)	34.6 (8.84)	243 (128)	315 (163)
AUC_0-t_ (hr*μg/mL)	149 (54.7)	657 (203)	484 (100)	2780 (862)	1670 (479)	9450 (2850)	8100 (3740)	35600 (15600)
t_1/2,z_ (day)	NC	7.9 (1.3)	NC	9.8 (1.6)	NC	10 (2.4)	NC	12 (2.4)
CL/F or CL (mL/hr)	NC	79.7 (38.2)	NC	78.2 (30.4)	NC	59.9 (16.3)	NC	34.0 (18.6)
AR	2.21 (0.74)		2.21 (0.52)		2.76 (0.89)		2.31 (0.34)	
Antibodies, n (%)								
Pre-dose only	0 (0)		1 (8)		1 (8)		1 (8)	
Post-dose only	7 (58)		1 (8)		1 (8)		6 (50)	
Pre- and post-dose	0 (0)		1 (8)		1 (8)		0 (0)	
Neutralizing	2 (17)		1 (8)		0 (0)		0 (0)	

*Abbreviations*: *AR* accumulation ratio, *AUC*_*0-t*_ plasma-concentration curve from time of treatment to the last measurable concentration, *C*_*max*_ maximum observed serum concentration, *C*_*0*_ estimated initial concentration after IV bolus dosing, *CL* clearance (CL*), CL/F* apparent clearance, *IV* intravenous, *NA* not applicable, *NC* not calculated, *SC* subcutaneous, *SD* standard deviation, *t*_*1/2,z*_ half-life, *t*_*max*_ time to C_max_

After single and four weekly SC injections, the median t_max_ ranged from 47 to 49 hours post-dose, indicating slow absorption with SC dosing (Tables 3, 4; Fig. 1b, c). Based on AUC and C_max_, exposure to blisibimod increased in an approximate dose-proportional manner from 0.3 to 3.0 mg/kg SC (Table 3). After a single dose, the mean t_½,z_ over this tenfold dose range was 8.5 days (Table 3); after the fourth weekly dose, 9.3 days (Table 4). The mean apparent CL/F was 85.1 mL/hr after a single dose and 72.9 mL/hr after multiple doses. Following four weekly doses of blisibimod, moderate accumulation was seen, with ratios ranging from 2.2 to 2.8 (Table 4).

### Anti-blisibimod antibodies

In the phase 1a study, 19 of the 40 (48 %) blisibimod- and four of the 13 (31 %) placebo-treated subjects were positive for anti-blisibimod binding antibodies at any time; none of these antibodies were neutralizing. In the phase 1b study, 19 of the 49 (39 %) blisibimod- and seven of the 14 (50 %) placebo-treated subjects were positive for anti-blisibimod binding antibodies at any time point; three (6 %) blisibimod-treated subjects were positive for neutralizing antibodies in this study. All subjects were negative for neutralizing antibodies at follow-up visits at the end of the study.

### Changes in lymphocyte populations following treatment with blisibimod

**Fig. 2:**
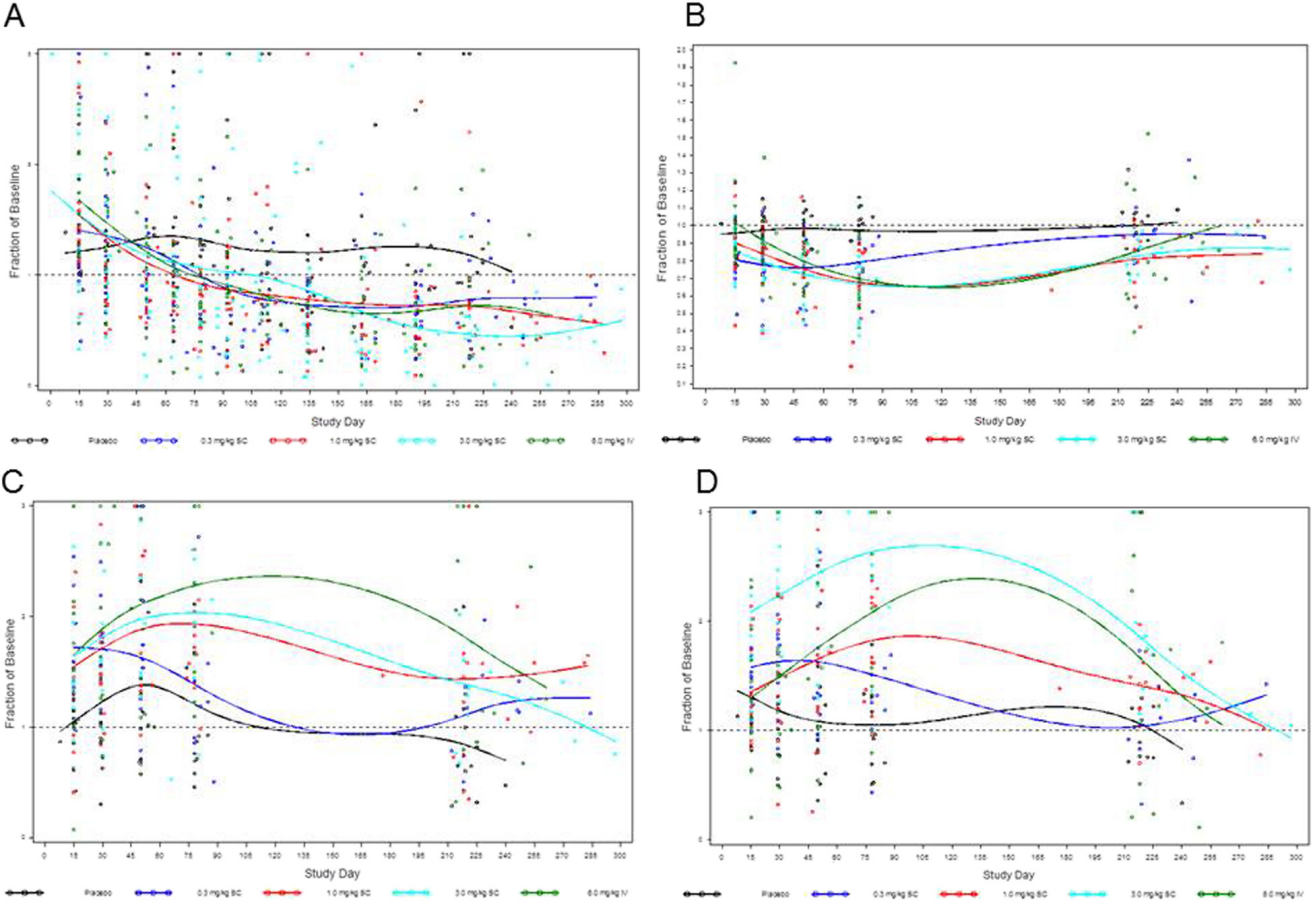
B cell subsets following administration of blisibimod to subjects with systemic lupus erythematosus treated with the four indicated weekly doses in the phase 1b study. B cell subsets are peripheral blood CD19^+^ CD20^+^ total B cells (**a**), IgD^+^ CD27^-^ naïve B cells (**b**), IgD^+^ CD27^+^ memory B cells (**c**), and IgD^-^ CD27^+^ memory B cells (**d**). Each symbol represents an individual patient at the indicated time point, and lines were generated by smoothing with a spline function

Baseline flow cytometry variables measured in this study were generally similar between the blisibimod and placebo groups (Table 5; Fig. 2a–d). Total lymphocytes, T cells, and natural killer cells did not exhibit significant changes relative to baseline. Three changes were observed with blisibimod: first, a reduction of total B cells (Fig. 2a, ≥ day 75; Table 5, day > 100, *P* = 0.006). Second, disproportionate changes occurred within the subsets of B cells that remained in the blood post-treatment: there was a decrease in the proportion of IgD^+^CD27^-^ naïve B cells (Fig. 2b, ≥ day 45; Table 5, day 43–100, *P* < 0.01) and an increase in the proportion of IgD^-^CD27^+^ memory B cells (Fig. 2d, > day 15; Table 5, day 15–42, *P* = 0.02). Third, there was an increase in the fluorescent intensity (i.e., antigen density) of CD19 (CD19 MFI, Table 5, > day 100, *P* < 0.01) and a decrease in the CD38 antigen on B cells (CD38 on B cells MFI, Table 5, day 15–42, *P* < 0.01).

**Table 5:** Lymphocyte counts and cell surface marker mean fluorescence intensity in phase 1b subjects with systemic lupus erythematosus (SLE) treated with blisibimod or placebo compared with previously established reference ranges in healthy subjects (HS)^b^ [27]

	Reference range^a^				Blisbimod (mg/kg)^a^				
Assay	HS	SLE^b^	CoR^c^	Placebo	0.3 SC	1.0 SC	3.0 SC	6.0 IV	*P* value^d^
Lymphocytes (mm^3^)	1915 (84)	1553 (129)^e^	ND						
Pre-dose^f^				1331 (700)	1653 (965)	1244 (458)	1677 (620)	1513 (707)	0.47
Day 15–42				1420 (61)	1632 (60)	1439 (59)	1408 (62)	1580 (59)	0.97
Day 43–100				1396 (74)	1390 (78)	1373 (72)	1357 (78)	1458 (74)	0.73
> Day 100				1432 (70)	1422 (71)	1286 (68)	1298 (73)	1305 (70)	0.10
T cells (mm^3^)	1544 (74)	1278 (109)	0.83						
Pre-dose^f^				1112 (628)	1275 (757)	965 (320)	1356 (547)	1141 (547)	0.51
Day 15–42				1086 (67)	1242 (67)	1150 (65)	1130 (72)	1247 (65)	0.52
Day 43–100				1086 (69)	1151 (73)	1121 (67)	1061 (73)	1232 (69)	0.95
> Day 100				1151 (55)	1205 (55)	1068 (53)	1094 (57)	1139 (55)	0.29
NK cells (mm^3^)	161 (18)	119 (14)	0.80						
Pre-dose^e^				75 (67)	87 (61)	120 (112)	103 (59)	124 (71)	0.46
Day 15–42				98 (12)	105 (12)	88 (12)	100 (13)	142 (12)	0.78
Day 43–100				116 (12)	112 (13)	99 (12)	119 (13)	112 (12)	0.67
> Day 100				108 (12)	106 (12)	102 (11)	112 (12)	115 (12)	0.92
CD19^+^CD20^+^ (mm^3^)	178 (17)	136 (15)	0.87						
Pre-dose^f^				112 (68)	194 (147)	140 (160)	169 (172)	165 (155)	0.70
Day 15–42				140 (21)	213 (21)	150 (20)	146 (22)	194 (20)	0.76
Day 43–100				126 (17)	132 (18)	98 (17)	96 (18)	115 (17)	0.17
> Day 100				111 (15)	117 (15)	70 (14)	60 (15)	66 (15)	0.01
IgD^+^CD27^+^ (naïve, mm^3^)	123 (15)	101 (14)	0.88						
Pre-dose^f^				73 (47)	138 (141)	110 (150)	131 (154)	91 (88)	0.70
Day 15–42				97 (14)	134 (15)	91 (14)	84 (15)	154 (14)	0.60
Day 43–100				89 (13)	82 (14)	53 (13)	32 (14)	69 (13)	< 0.01
> Day 100				75 (12)	82 (13)	49 (11)	41 (13)	31 (13)	0.06
IgD^+^CD27^+^ (memory, mm^3^)	18.9 (2.0)	12.0 (2.2)^e^	0.80						
Pre-dose^f^				10 (13)	13 (11)	7 (7)	9 (9)	6 (5)	0.44
Day 15–42				9 (3)	23 (3)	14 (3)	14 (3)	23 (3)	0.09
Day 43–100				8 (3)	14 (4)	14 (3)	11 (4)	25 (3)	0.31
> Day 100				7 (2)	7 (2)	7 (2)	5 (2)	7 (2)	0.67
IgD^-^CD27^+^ (memory, mm^3^)	24.5 (2.5)	14.1 (2.2)^g^	0.92						
Pre-dose^f^				17 (17)	20 (11)	15 (11)	11 (7)	14 (10)	0.40
Day 15–42				15 (4)	37 (4)	24 (3)	28 (4)	32 (4)	0.02
Day 43–100				14 (4)	23 (4)	19 (4)	21 (4)	34 (4)	0.22
> Day 100				11 (3)	16 (3)	10 (3)	10 (3)	12 (3)	0.73
CD19 (MFI)	63.0 (1.7)	44.6 (2.7)^g^	ND						
Pre-dose^f^				70 (37)	44 (11)	68 (22)	57 (9)	76 (17)	< 0.01
Day 15–42				69 (5)	68 (5)	75 (4)	76 (5)	74 (5)	0.19
Day 43–100				65 (7)	73 (8)	83 (6)	81 (7)	80 (7)	0.04
> Day 100				66 (5)	68 (5)	81 (4)	82 (5)	43 (5)	<0.01
CD38 (on B cells; MFI)	295 (18)	486 (52)^g^	ND						
Pre-dose^f^				578 (477)	413 (175)	424 (374)	498 (197)	537 (292)	0.68
Day 15–42				608 (67)	362 (67)	384 (64)	363 (71)	352 (65)	< 0.01
Day 43–100				625 (96)	386 (101)	607 (92)	397 (100)	463 (96)	0.30
> Day 100				477 (104)	421 (104)	879 (99)	654 (106)	439 (104)	0.02

*Abbreviations*: *CoR* coefficient of reliability, *IV* intravenous, *MFI* median fluorescence intensity, *ND* not determined, *NK* natural killer, *SC* subcutaneous, *SD* standard deviation, *SE* standard error, *SLE* systemic lupus erythematosus

^a^Table values are mean (SE) for reference ranges, mean (SD) for pre-dose values and least squares mean (SE) for post-dose study periods

^b^Reference range *P* values compare values from patients with mild-to-moderate SLE with those of HS

^c^CoR was calculated by dividing the inter-subject variability by the total variability (inter-subject + intra-subject variability). This provides a reference to assess the reliability of a measure in detecting changes within a subject over time. A higher CoR indicates a measure that will be more reliable in detecting treatment effects over time since the within (intra-) subject variability is low in repeat measures testing

^d^Blisibimod 1.0 mg/kg and 3.0 mg/kg dose groups were pooled and compared to placebo using an F-test to generate descriptive *P* values

^e^ *P* value from 0.01 to < 0.05

^f^Pre-dose is defined as the mean value of day 1 and the immediately preceding screen

^g^ *P* value < 0.01

### Safety and tolerability

In the phase 1a study, 28 (70 %) blisibimod- and 11 (79 %) placebo-treated subjects reported ≥ 1 AE (Table 6). Two SAEs were reported: one each (8 % each) in the blisibimod 3.0 mg/kg SC (17 %; ureteral stones, hydronephrosis, and pyelonephritis; this subject continued in the study) and in the placebo cohorts (7 %; pneumococcal pneumonia and bacteremia; this subject withdrew from the study). Both SAEs were considered by the investigator to be related to the study medication.

**Table 6:** Adverse events (AE) occurring in ≥ 5 % of patients with systemic lupus erythematosus treated with blisibimod in the phase 1a study

		Blisibimod							
	Placebo	mg/kg SC				mg/kg IV			All
n (%)	(n = 14)	0.1 (n = 6)	0.3 (n = 6)	1.0 (n = 9)	3.0 (n = 6)	1.0 (n = 6)	3.0 (n = 1)	6.0 (n = 6)	(N = 40)
Patients reporting an AE	11 (79)	5 (83)	3 (50)	4 (44)	5 (83)	5 (83)	1 (100)	5 (83)	28 (70)
Nausea	4 (29)	2 (33)	0 (0)	2 (22)	2 (33)	0 (0)	0 (0)	0 (0)	6 (15)
Headache	3 (21)	1 (17)	0 (0)	1 (11)	1 (17)	0 (0)	0 (0)	1 (17)	4 (10)
Upper respiratory tract infection	3 (21)	0 (0)	0 (0)	1 (11)	0 (0)	1 (17)	1 (100)	1 (17)	4 (10)
Diarrhea	1 (17)	0 (0)	0 (0)	1 (11)	1 (17)	0 (0)	0 (0)	1 (17)	3 (8)
Bronchitis	0 (0)	0 (0)	0 (0)	0 (0)	0 (0)	0 (0)	1 (100)	1 (17)	2 (5)
Dizziness	4 (29)	0 (0)	0 (0)	1 (11)	1 (17)	0 (0)	0 (0)	0 (0)	2 (5)
Injection site prutitus	1 (7)	2 (33)	0 (0)	0 (0)	0 (0)	0 (0)	0 (0)	0 (0)	2 (5)
Pain in extremity	1 (7)	0 (0)	0 (0)	0 (0)	1 (17)	0 (0)	0 (0)	1 (17)	2 (5)
Pharyngolaryngeal pain	0 (0)	0 (0)	0 (0)	0 (0)	0 (0)	2 (33)	0 (0)	0 (0)	2 (5)
Shoulder pain	0 (0)	2 (33)	0 (0)	0 (0)	0 (0)	0 (0)	0 (0)	0 (0)	2 (5)
Vomiting	1 (7)	0 (0)	0 (0)	1 (11)	1 (17)	0 (0)	0 (0)	0 (0)	2 (5)

*Abbreviations*: *IV* intravenous, *SC* subcutaneous

In the phase 1b study, 47 (96 %) blisibimod- and 12 (92 %) placebo-treated subjects reported ≥ 1 AE (Table 7). Nine SAEs were reported in eight (13 %) subjects (blisibimod, n = 5 across all dose groups [10 %]; placebo, n = 3 [23 %]). In the in the 0.3 mg/kg blisibimod SC cohort, two subjects experienced SAEs: one a polyarthritis lupus flare and the other severe depression (8 % each). In the blisibimod 1.0 mg/kg SC cohort, one subject experienced chest pain, pyrexia, and a moderate increase in prothrombin time; in the 3.0 mg/kg SC cohort, one subject experienced moderate syncope (8 % each). None of these SAEs were considered related to the study drug and all subjects remained in the study. In the blisibimod 6.0 mg/kg IV cohort, one (8 %) subject experienced SAEs of severe sick sinus syndrome and ECG QTc interval prolongation, which were considered by the investigator to possibly be related to the study drug; however, this subject remained in the study. In the placebo cohort, SAEs of pericarditis and pericardial effusion, mild SLE flare, and cellulitis were reported in three subjects (23 %); none were considered related to the study drug and all subjects remained in the study.

**Table 7:** Adverse events (AE) occurring in ≥ 10 % of patients with systemic lupus erythematosus treated with blisibimod in the phase 1b study

		Blisibimod				
	Placebo	mg/kg SC			6.0 mg/kg IV	All
n (%)	(n = 13)	0.3 (n = 12)	1.0 (n = 13)	3.0 (n = 12)	(n = 12)	(N = 49)
Patients reporting an AE	12 (92)	12 (100)	13 (100)	11 (92)	11 (92)	47 (96)
Nasopharyngitis	1 (8)	2 (17)	5 (38)	2 (17)	2 (17)	11 (22)
Headache	0 (0)	0 (0)	2 (15)	2 (17)	2 (17)	11 (22)
Injection site erythema	0 (0)	0 (0)	2 (31)	3 (25)	0 (0)	7 (17)
Nausea	1 (8)	1 (8)	1 (8)	3 (25)	2 (17)	7 (14)
Injection site prutitus	0 (0)	0 (0)	3 (23)	3 (25)	0 (0)	6 (12)
Upper respiratory tract infection	0 (0)	3 (25)	2 (15)	0 (0)	1 (8)	6 (12)
Urinary tract infection	2 (15)	0 (0)	2 (15)	3 (25)	1 (8)	6 (12)
Arthralgia	2 (15)	1 (8)	1 (8)	2 (17)	1 (8)	5 (10)
Back pain	0 (0)	1 (8)	1 (8)	2 (17)	1 (8)	5 (10)
Diarrhea	3 (23)	1 (8)	1 (8)	3 (25)	0 (0)	5 (10)
Gastroesophogeal reflux disease	0 (0)	1 (8)	2 (15)	2 (17)	0 (0)	5 (10)
Systemic lupus	2 (15)	2 (17)	1 (8)	1 (8)	1 (8)	5 (10)

*Abbreviations*: *IV* intravenous, *SC* subcutaneous

One (8 %) subject in the phase 1b blisibimod 3.0 mg/kg SC group had a significant change from baseline in Fridericia QTc interval; no other clinically meaningful changes in ECGs were seen. No clinically significant drug-related trends were observed in clinical laboratory data or vital signs.

## Discussion

In the present studies, treatment with blisibimod produced significant changes in B cell subpopulations and B cell surface molecule density were observed, with no notable changes in T cells or natural killer cells. There was an initial transient increase of the B cell pool in subjects treated with single or multiple doses of blisibimod, but this was not statistically significant and was similar to previous findings in SLE patients treated with the BAFF antagonists atacicept [29, 30] and belimumab [31, 32]. Blisibimod showed consistency in half-life and linearity across dose ranges for both IV and SC routes of administration, with half-life ranging from 7 to 12 days for the 0.3 to 6.0 mg/kg IV and SC dose groups, and clearance estimates constant over the 1.0 to 6.0 mg/kg IV and SC doses ranging from 34 to 79 mL/hr. The safety and tolerability of single or multiple SC or IV doses of blisibimod were comparable with those associated with placebo administration, and AE and treatment-related events did not increase with increasing doses of blisibimod.

The main finding of these studies was the statistically significant decrease in B cell counts (Table 5, > day 100, *P* = 0.10), although the reduction was not as large as those seen during treatment with the lytic agent rituximab [33]. The constituency of the B cell pool also changed following treatment with blisibimod: there was a decrease in the number of naïve B cells (24–76 % reduction, day 43–100) and a relative increase in the memory B cell compartment, with the greatest effect on IgD^-^CD27^+^ (53–154 % increase, day 15–42). One possible mechanism for these results may be related to differences in the expression profiles of BAFF receptors on various B cell subsets, as BAFF is known to differentially support the stages of B cell development [1–5]. The relative increase in the memory B cell compartment was not associated with safety signals or elevations of immunoglobulin or anti-blisibimod antibody levels. In fact, when comparing these data with those of healthy volunteers from our previous study [27], blisibimod seemed to correct the B cell compartment to be more like that of healthy subjects, suggesting that if normalization of these cell types can be associated with clinical improvement, these may be a treatment goal in future studies.

Markers of the metabolic activity of targeted cells were also examined, as BAFF deprivation might suppress excess metabolic activity of those cells that were not depleted. The fluorescence intensity of CD19 was measured because CD19 is part of the signaling complex that modulates the threshold of activation of the B cell receptor, and it is known to exhibit genetic variations that correlate to expression levels and autoimmunity [34]. The changes in CD19 density were variable, with increases of 119–154 % for all doses except 6.0 mg/kg IV, which produced a 43 % reduction > day 100 (Table 5, CD19 MFI day 43–100 and day > 100, *P* = 0.04 and *P* < 0.01, respectively). However, interpretation of these results is difficult because the CD19 MFI values at baseline were statistically significantly different between the blisibimod- and placebo-treated groups (Table 5; CD19 on B cells MFI, pre-dose, *P* < 0.01). The variability in this dataset may be due to inconsistencies in the laboratory methods that were used; however, as the coefficient of reliability from our validation exercises all exceeded 0.8, it is more likely attributable to biologic variability in our subject pool. Nonetheless, CD19 MFI was increased during administration of blisibimod, raising the possibility that blisibimod promoted a reduction in B cell activation that reversed the down-modulation of CD19 from the B cell surface in SLE patients [35]. Reduction of CD38 was also seen in blisibimod-treated subjects, occurring as early as day 15–42 of treatment (Table 5; CD38 on B cells MFI, day 15–42, *P* < 0.01). This brought the density of this marker to within the range of healthy volunteers (362–384 vs. 295 CD38 on B cells MFI).

Other agents that reduce the number of circulating B cells have been tested in SLE: the anti-CD20 monoclonal antibody rituximab, a targeted B cell depletor, has been used in the clinical setting in patients with SLE despite not being approved for use in this population [36, 37]. The anti-BAFF monoclonal antibody belimumab in two phase 3 trials in SLE demonstrated improvements in clinical measurements and biomarkers of disease activity [22, 23], and is approved for the treatment of SLE in the United States. The BAFF- and a proliferation-inducing ligand (APRIL)-neutralizing atacicept also reduces B cell counts in patients with SLE, underscoring the versatility of options in modulating this pathway [29, 38]. The success of belimumab and the absence of efficacy above standard of care from the rituximab clinical trials may be multifactorial and may dependent on trial design, but these results could suggest that efficacy in treating SLE may not solely be a function of reductions in B cell numbers. Yet, the positive results seen with belimumab confirm the possibility of therapeutic potential of BAFF antagonists in SLE.

A key question is whether agents that antagonize both cell-bound and circulating BAFF (e.g., blisibimod or tabulumab) will have a different spectrum of efficacy than those that bind only to soluble BAFF (e.g., belimumab) [39]. In a 24-week, phase 2b, placebo-controlled study of blisibimod in 547 patients with SLE, treatment with blisibimod decreased B cell counts, reduced disease activity, and prolonged time to first severe disease flare [40]. The reductions in B cell counts in this study were comparable with those reported with belimumab [23]. Likewise, a preliminary report confirmed some B cell reductions in phase 3 trials of the soluble and bound BAFF antagonist tabulumab [41].

Little is known about the nature and purpose of membrane-bound BAFF. The phenotype of genetically engineered mice that express membrane-bound BAFF and almost no soluble BAFF is similar to that of BAFF-deficient mice, with marked reductions in B cells, serum immunoglobulin (Ig) levels, and antigen-specific Ig responses [42]. Based on this, patients with SLE in whom both membrane-expressed and soluble BAFF have been neutralized may have a clinical response no different from that of patients in whom only soluble BAFF has been neutralized (e.g., belimumab). However, since expression of membrane-bound BAFF in the genetically engineered mice was low [42], there may not have been sufficient membrane-expressed BAFF to clarify its function.

In addition to effects on B cells, BAFF can co-stimulate in vitro proliferation of and cytokine production by T cells [43, 44], and in mice it can skew in vivo inflammatory responses toward a T helper (Th) 1 cell profile and away from a Th2 cell profile in a B cell-independent manner [45]. Moreover, BAFF promotes generation of Th17 cells at least in part through direct effects on T cells [46]. Whether membrane-expressed BAFF might contribute to these BAFF-driven effects on T cells is unknown.

These were a priori first-in-human studies, and the reason for the large portion of subjects testing positive for neutralizing antibodies is unknown. Ten and 4 % of subjects in the phase 1a and 1b studies, respectively, tested positive for antibodies prior to dosing. As previous use of an investigational product was an exclusion criteria (belimumab was an investigational product at the time of the studies), prior exposure to belimumab would not explain these findings. Of the three subjects who had neutralizing antibodies during the studies, the nature and frequency of adverse events did not differ from the group as a whole, and all three subjects were negative for neutralizing antibodies at the end of the study. Given that a large portion of the subjects in these studies were positive for antibodies at any time, we believe this may be a characteristic of this population or perhaps a limitation of the assay, which may require further development [40].

A limitation of these studies is that this population had mild stable or inactive disease (mean SLE Disease Activity Index score of approximately 3) and baseline immune-phenotypic profiles that were more similar to those of the healthy population than patients with SLE [27]. This may have made it difficult to appreciate significant immunologic changes seen in these studies. Moreover, many patients were being treated with immunosuppressives and steroids, which could have impacted baseline B cell levels. There were also some baseline differences between the dosing groups in some B cell measurements. Although this most likely reflects the heterogeneity of the population, this may have muted or amplified treatment responses in these small treatment groups.

## Conclusions

Single and multiple doses of blisibimod produced approximate dose-proportional pharmacokinetics across the dosing range of 1.0–6.0 mg/kg, and significant changes in B cell subpopulations and B cell surface molecule density. Blisibimod was well tolerated, with a safety and tolerability profile similar to that of placebo. These findings support the potential of BAFF antagonists such as blisibimod in the treatment of SLE and other B cell-mediated diseases. Larger clinical trials of blisibimod in SLE should be advanced to assess its efficacy in SLE and other autoimmune diseases in which B cells play an important role in pathogenesis.

## Abbreviations

AE: adverse event
APRIL: a proliferation-inducing ligand
AR: accumulation ratio
AUC_0-t_: area under the plasma-concentration curve from time of treatment to the last measureable concentration
BAFF: B cell-activating factor
BLyS: B lymphocyte stimulator
C_0_: estimated initial concentration after IV bolus dosing
CL: clearance
CL/F: apparent clearance after SC dosing
C_max_: maximum observed serum concentration
ECG: electrocardiogram
IC_50_: half-maximal inhibitory concentration
Ig: immunoglobulin
IRB: Institutional Review Board
IV: intravenous
MedDRA: Medical Dictionary for Regulatory Activities
MFI: median fluorescence intensity
NIH CTCAE: National Institute of Health Common Toxicity Criteria for Adverse Events
SAE: serious AE
SC: subcutaneous
SLE: systemic lupus erythematosus
t_1/2,z_: half-life
Th: T helper cell
t_max_: time to C_max_
